# Role of the E3 Ubiquitin Ligase TRIM4 in Predicting the Prognosis of Hepatocellular Carcinoma

**DOI:** 10.7150/jca.37164

**Published:** 2020-04-06

**Authors:** Zhao-Ru Dong, Wei Zhou, Dong Sun, Yu-Chuan Yan, Chun-Cheng Yang, Ya-Fei Yang, Hai-Chao Li, Xu-Ting Zhi, Tao Li

**Affiliations:** Department of General Surgery, Qilu Hospital, Shandong University, Jinan 250012, China

**Keywords:** E3 ubiquitin ligase, TRIM4, hepatocellular carcinoma, progression

## Abstract

The E3 ubiquitin ligase TRIM4 has been reported to regulate the assembly of the antiviral signalling complex, induce mitochondrial aggregation and sensitize cells to H_2_O_2_-induced death. However, the relationship between TRIM4 and human malignancies, including hepatocellular carcinoma (HCC), is unclear. In this study, we detected the expression of TRIM4 in 134 pairs of HCC tissues and peritumoural tissues and investigated the association of TRIM4 expression with the prognosis of HCC. We found that the TRIM4 expression was much lower in HCC tissues than in peritumoural tissues and was significantly associated with vascular invasion, tumour capsule and Hong Kong Liver Cancer (HKLC) stage. Univariate and multivariate analyses revealed that the TRIM4 expression was an independent prognostic factor for overall survival (OS) and recurrence-free survival (RFS) in our HCC cohort. Patients with higher TRIM4 expression had a lower incidence of intrahepatic recurrence and a higher OS rate (*p*<0.001 and *p*<0.01, respectively). These results were further validated in another independent cohort of 200 HCC patients. In conclusion, the TRIM4 level in HCC tissues is an independent prognostic factor for HCC patients. Close clinical monitoring is recommended for patients with low TRIM4 expression.

## Introduction

Ubiquitination is a multistep enzymatic process that results in the attachment of ubiquitin, or chains of ubiquitin, to the target protein. Previous studies have confirmed that ubiquitination is an important posttranslational modification and is involved in the regulation of a number of intracellular events, including transcription, the cell cycle, apoptosis, tumorigenesis and development 1-4. The biochemical reaction of ubiquitination requires several essential enzymes, including E1, E2 and E3 enzymes. Among these enzymes, E3 ubiquitin ligases are regarded as receptors for recognizing specific target proteins 1-5.

Tripartite motif (TRIM)-containing proteins serve an important function as E3 ubiquitin ligases. Generally, TRIM proteins are defined by the presence of an N-terminal RING finger, one or two B-boxes and a coiled-coil domain. A RING domain catalyses ubiquitin chain formation on its substrate via binding to a ubiquitin-conjugating enzyme (E2). A B-box domain may also exhibit E3 ligase activity in certain TRIM proteins that lack a functional RING domain. TRIM genes have been subclassified based on differences in their C-terminal domains 6-8. Recent studies have shown that several TRIM proteins are involved in the tumorigenesis and development of solid malignancies, including hepatocellular carcinoma (HCC) 9-10. For example, TRIM24 can function as a liver-specific tumour suppressor in mice and co-regulate hepatocarcinogenesis in an antagonistic manner with Rara 9. In addition, TRIM16 has been proven to inhibit cell migration, invasion and epithelial-mesenchymal transition in HCC to suppress tumour progression 10.

TRIM4 is a member of the TRIM family and its cellular function remains unclear. TRIM4 has been shown to regulate the assembly of the mitochondrial antiviral signalling complex and K63-linked ubiquitination of RIG-1 11. Recently, TRIM4 has also been reported to transiently interact with mitochondria, induce mitochondrial aggregation and sensitize HSK293T cells to H_2_O_2_-induced cell death 12. However, little is known about the biological effects of TRIM4 on HCC. In this study, we detected the expression of TRIM4 in HCC tissues and corresponding peritumoural tissues and investigated the association of TRIM4 expression with HCC patient's prognosis. Our data demonstrate that decreased TRIM4 expression is significantly associated with HCC progression and worse patient survival and is an independent prognostic factor for postoperative recurrence.

## Material and Methods

### Patients and Tissue Samples

This study was conducted with the approval of the Ethics Committee of Qilu Hospital, Shandong University (Jinan, China). Informed consent was obtained from each patient. Eight paired frozen HCC and adjacent non-tumour liver tissue samples were collected from patients who underwent curative resection at Qilu Hospital, Shandong University, between January 2010 and December 2012. Archival specimens used for tissue microarrays (TMAs) were obtained from HCC patients who underwent curative resection. The inclusion and exclusion criteria of the patient cohorts included (a) having a distinctive pathologic diagnosis of HCC, (b) having no anticancer treatment before liver resection, (c) having curative liver resection, (d) having suitable formalin-fixed, paraffin-embedded tissues and (e) having complete clinicopathologic and follow-up data. All patients were followed regularly in the outpatient clinic and were monitored prospectively for recurrence according to a standard protocol as previously described 13-15.

### Western Blot Analysis

Proteins extracted from tissues were subjected to sodium dodecyl sulfate-polyacrylamide gel electrophoresis, transferred onto polyvinylidene fluoride membranes, and incubated with a TRIM4 antibody and a secondary antibody. Proteins were visualized using HRP-conjugated IgG and Immobilon Western Chemiluminescent HRP Substrate (Millipore).

### Construction of Tissue Microarrays and Immunohistochemistry

Tissue microarray (TMA) was constructed as described in our previous studies 13-15. Immunohistochemical staining for TRIM4 was performed on formalin-fixed sample sections from the TMA. The slides were dewaxed and washed three times with xylene. Tissues were then rehydrated, and endogenous peroxidase activity was blocked. After antigen retrieval, the TMA slides were incubated in 5% normal goat serum at room temperature to reduce nonspecific reactions. Subsequently, the slides were incubated overnight at 4°C with rabbit monoclonal anti-TRIM4 antibody, incubated with secondary antibody, and then stained with diaminobenzidine (DAB)-H_2_O_2_. Finally, the TMA slides were counterstained with haematoxylin, dehydrated, and mounted with a coverslip using standard medium. Phosphate buffer solution was used instead of the primary antibody as a negative control.

### Evaluation of Immunostaining Intensity

TRIM4 was immunohistochemically stained in the cell cytoplasm. Cases were scored independently based on the intensity of cellular staining and the proportion of stained tumour cells. The product of the intensity and proportion scores was used to determine the level of TRIM4, as described in our previous studies 13, 14.

### Statistical Analysis

Fisher's exact test or χ^2^ test was used to evaluate relationships between TRIM4 expression and clinicopathological parameters of patients with HCC. The relative prognostic significance of variables for predicting OS and RFS were analysed using Cox proportional hazards regression models. All *p*-values were two-sided, and *p*<0.05 was regarded as statistically significant. All statistical analyses were performed using SPSS 21.0 for Windows (SPSS, Chicago, IL).

## Results

### TRIM4 Expression in Human HCC Tissues

**Figure 1:**
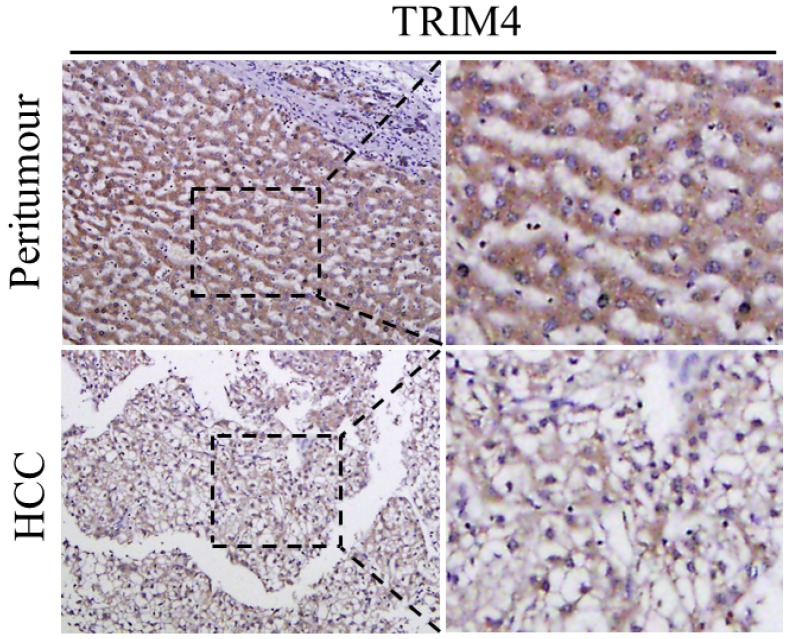
Immunohistochemical analysis of TRIM4 expression in HCC and peritumoural tissues.

**Figure 2:**
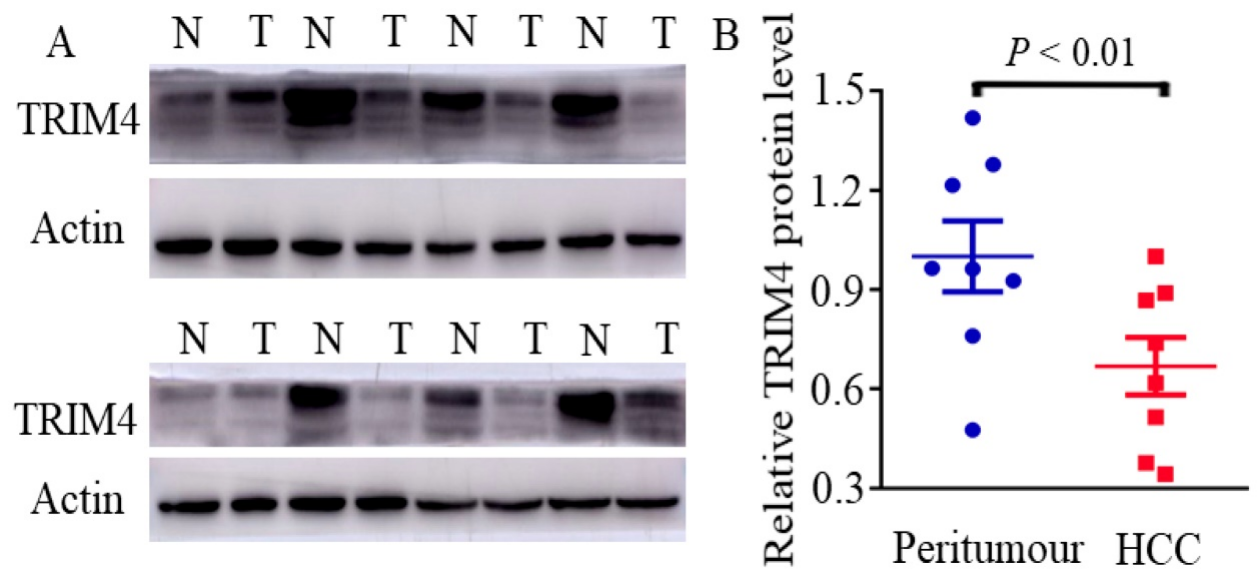
Immunoblotting analysis of TRIM4 levels in HCC and peritumoural tissues. (N: peritumoural tissue; T: HCC tissue)

First, immunohistochemical staining was used to detect TRIM4 expression in eight human HCC tissues and corresponding peritumoural tissues. Our results showed that TRIM4 expression was much lower in human HCC tissues than in corresponding peritumoural tissues (Figure 1). Second, western blotting was performed to determine TRIM4 protein levels in these samples. These findings, in accordance with the immunohistochemical staining results, also revealed that TRIM4 levels were significantly lower in human HCC tissues than in peritumoural tissues (*p*<0.01, Figure 2A and B).

### Clinicopathological Features of TMAs

We further used immunohistochemistry and the TMA technique to evaluate TRIM4 levels in human HCC tissues. In our present study, samples from 134 primary HCC cases were stained for TRIM4. Individuals in the patient cohort, which consisted of 115 males and 19 females, had a median age of 57 years (range: 26 to 74 years) at the time of surgery. There were 109 HBsAg-positive patients and 25 HBsAg-negative patients; all of the patients were negative for HCV. Patients were histopathologically categorized according to the Hong Kong Liver Cancer (HKLC) staging system. An early-stage tumour was defined as a tumour with diameter ≤5 cm, ≤3 tumour nodules and no intrahepatic venous invasion 16. Tumours beyond the early stage were categorized as advanced-stage tumours. There were 57 early-stage tumours and 77 advanced-stage tumours based on the HKLC staging system.

### Relationship between TRIM4 Expression and the Clinicopathological Parameters of HCC Patients

**Figure 3:**
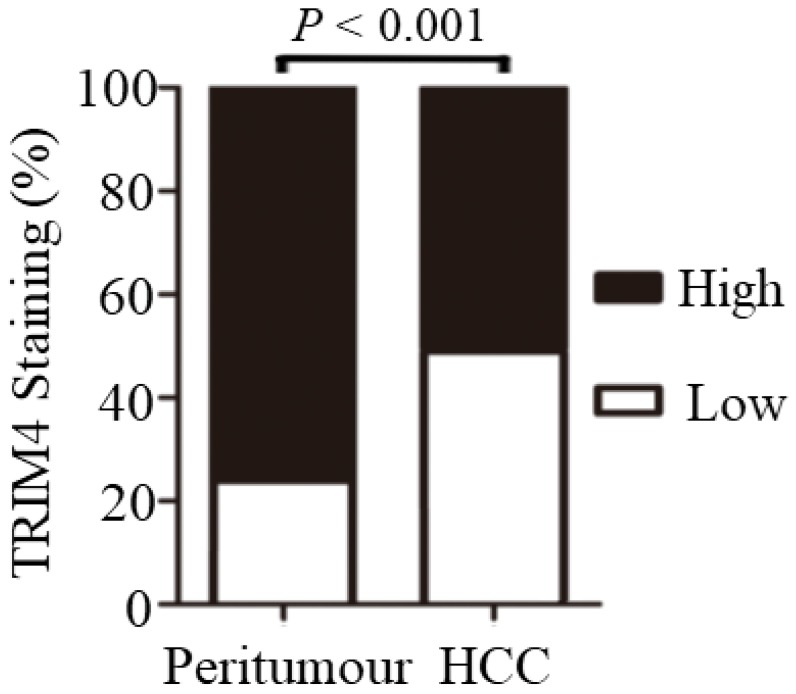
Immunohistochemical analysis revealed that TRIM4 expression was much lower in HCC tissues than in matched peritumoural tissues

Significant differences in TRIM4 staining patterns were generally observed between tumour-adjacent tissues and primary HCC tissues. Statistical analysis revealed that TRIM4 expression was much lower in HCC tissues than in matched peritumoural tissues (*p*<0.001, Figure 3).

**Figure 4:**
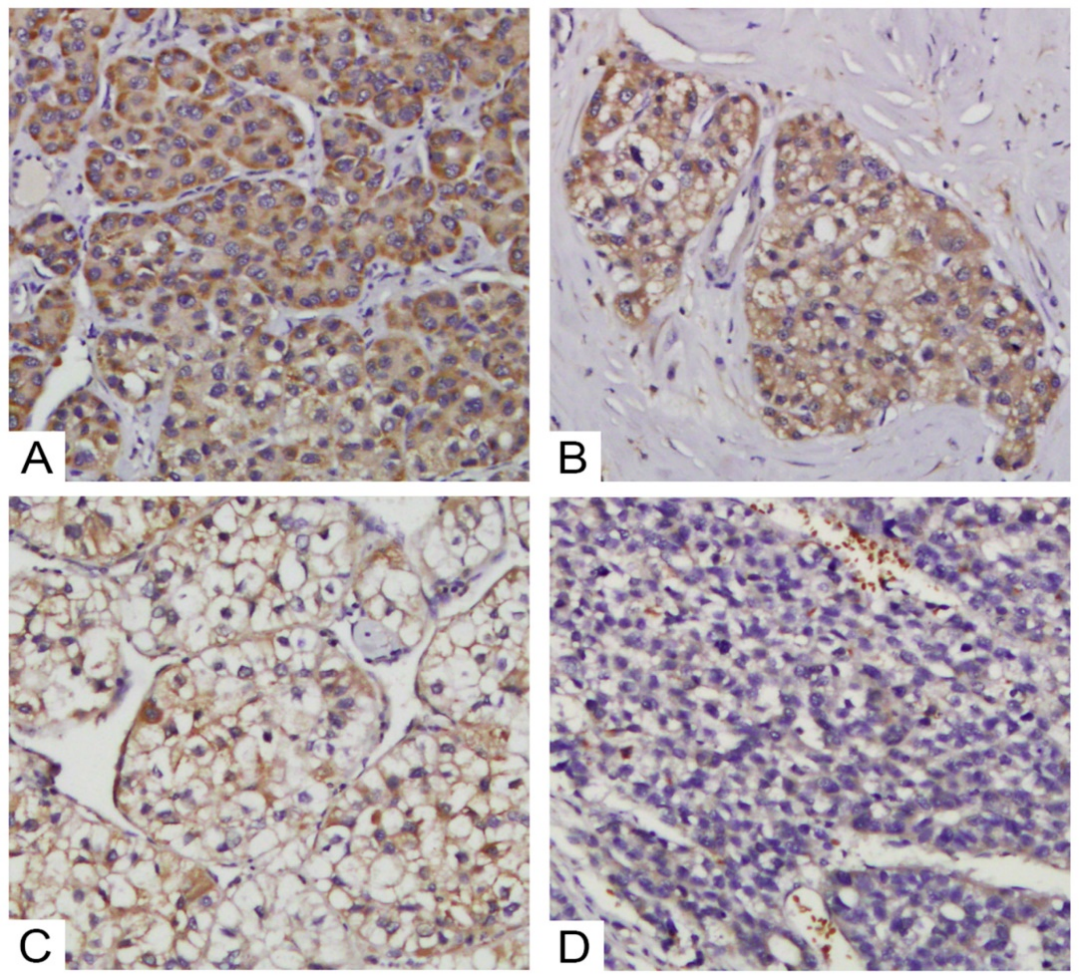
Various levels of TRIM4 staining were observed in HCC tissues. Representative images of staining in tumour samples: high expression (A and B) and low expression (C and D).

We then further investigated the correlations between TRIM4 expression and HCC patients' clinicopathological parameters. Based on TRIM4 staining scores (Figure 4), we divided our HCC cohort into the high TRIM4 cohort and the low TRIM4 cohort. Our results suggested that the TRIM4 level was associated with vascular invasion (*p*=0.029), tumour capsule (*p*<0.01) and HKLC stage (*p*=0.034); however, no significant associations were detected between TRIM4 expression and age, sex, tumour size, hepatitis B virus (HBV) infection, tumour number, cirrhosis, grade of tumour differentiation or serum alpha-fetoprotein (AFP) concentration (Table 1).

### Correlation of TRIM4 Expression with the RFS and OS of HCC Patients

**Figure 5:**
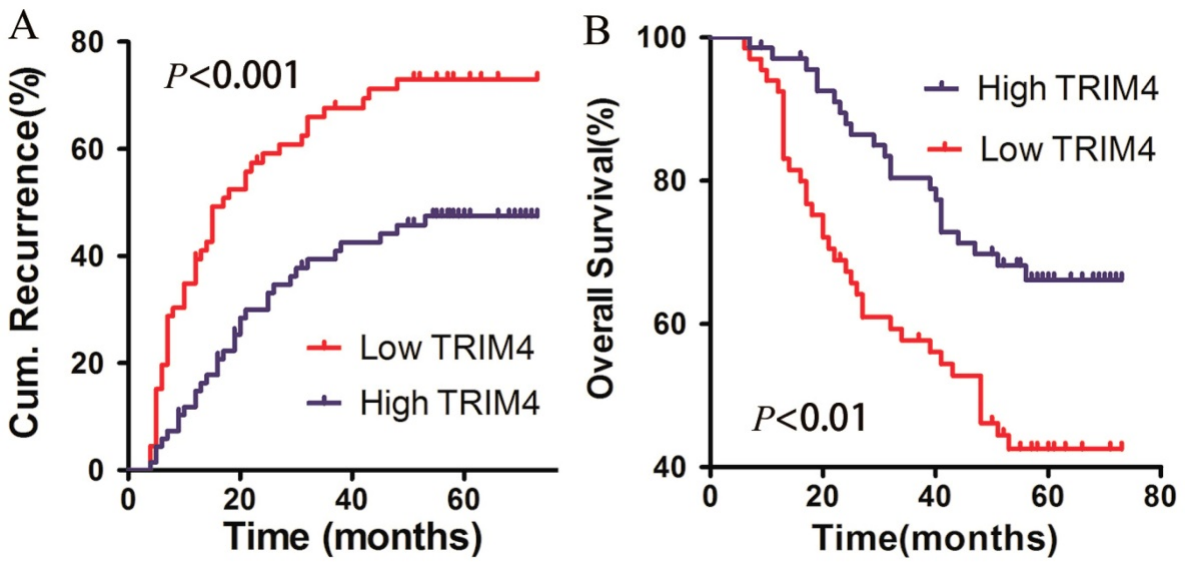
Kaplan-Meier curves for intrahepatic recurrence (A) and overall survival (B), comparing HCC patients with high and low TRIM4 levels.

To determine the association between TRIM4 protein levels in HCC tissues and patient prognosis, all patients in our cohort were followed up to determine OS and RFS after surgery. The 1-, 2-, and 5-year cumulative incidences of intrahepatic recurrence in the high TRIM4 cohort were 14.78%, 29.99%, and 47.45%, respectively, while the 1-, 2-, and 5-year cumulative incidences of intrahepatic recurrence in the low TRIM4 cohort were 39.39%, 59.12%, and 73.03%, respectively. The high and low TRIM4 cohorts had significantly different cumulative incidences of intrahepatic recurrence (log-rank test, χ2=12.190, *p*<0.001, Figure 5A). The 1-, 2-, and 5-year OS rates of the high TRIM4 cohort were 97.04%, 87.94%, and 66.16%, respectively, significantly better than those of the low TRIM4 cohort (92.42%, 67.32%, and 42.52%, respectively) (χ2=8.874, *p*<0.01, Figure 5B).

### Multivariate Analyses of Predictive Factors for Intrahepatic Recurrence

Univariate analysis revealed that TRIM4 expression, tumour diameter, vascular invasion and tumour capsule were predictive factors for intrahepatic recurrence. Multivariate analysis revealed that TRIM4 expression (hazard ratio, 0.566; 95% CI, 0.345-0.930; *p*=0.025), tumour diameter (hazard ratio, 1.804; 95% CI, 1.131-2.876; *p*=0.013) and vascular invasion (hazard ratio, 1.802; 95% CI, 1.115-2.912;* p*=0.016) were independent predictive factors for RFS (Table 2).

### Multivariate Analyses of Predictive Factors for the OS of HCC Patients

Univariate analyse revealed that TRIM4 expression, tumour diameter, and vascular invasion were the factors that significantly influenced the OS of HCC patients. Subsequently, the multivariate analyses further revealed that the TRIM4 level (hazard ratio, 0.550; 95% CI, 0.321-0.944; *p*=0.030), tumour diameter (hazard ratio, 2.191; 95% CI, 1.264-3.798; *p*<0.01), and vascular invasion (hazard ratio, 2.249; 95% CI, 1.322-3.826; *p*<0.01) were independent predictive factors for OS of HCC patients (Table 3).

### Validation

We next validated the prognostic value of TRIM4 in another independent cohort of 200 HCC patients who underwent hepatectomy and had a median age of 50 years. The results we obtained were similar to those acquired for the training set. Patients with lower TRIM4 levels had a higher cumulative incidence of intrahepatic recurrence and a lower OS rate in this cohort of HCC patients. Multivariate analyses also confirmed that TRIM4 expression was an independent predictor of RFS and OS (Tables 4 and 5).

## Discussion

As the third leading cause of cancer mortality worldwide, HCC is one of the most prevalent malignancies and remains a major public health challenge. Partial hepatectomy is the most common curative treatment for HCC 17, 18, but the high incidence of intrahepatic recurrence and metastasis is the major challenge that must be overcome in order to improve the prognosis of HCC patients undergoing surgery. Therefore, it is important to identify predictive factors for HCC recurrence and metastasis to ensure close monitoring for patients with high recurrence risk and then utilize individualized therapy strategies.

The role of E3 ubiquitin ligases is to mediate the chemical transfer of the C-terminus of ubiquitin from an E2 ubiquitin-conjugating enzyme to a protein substrate, and ubiquitinated substrates undergo different fates depending on the site and type of ubiquitination. Traditionally, E3 ubiquitin ligases have been categorized into two classes of RING/U-box ligases and HECT ligases. Both types of ligases have been reported to lead to positive or negative regulation of multiple cancer-related signal pathways and maybe potential novel targets for anticancer therapies 13, 19.

TRIM4, a member of the TRIM family with a unique domain organization that belongs to the RING family of ubiquitin ligases 20, 21, is a cytoplasmic E3 ubiquitin ligase; this localization differs from that of certain other TRIM family members, such as TRIM24, which has been verified to be a nuclear protein. Evidence has demonstrated that TRIM4 can sensitize HEK293 cells to H2O2-induced cell death. However, the role of TRIM4 in hepatocarcinogenesis and HCC development remains to be elucidated. This study indicated that TRIM4 expression was much lower in human HCC tissues than in peritumoural tissues. The OS rate was significantly higher for HCC patients with high TRIM4 expression than for HCC patients with low TRIM4 expression. These results reveal that TRIM4 can be a valuable biomarker for evaluating HCC prognosis and that the loss of the TRIM4 protein in HCC tissues consistently suggests a worse prognosis.

Numerous studies have revealed that vascular invasion is an independent risk factor for the prognosis of HCC patients who underwent surgical treatment 22-24. The presence of vascular invasion of the portal or hepatic veins is associated with a high risk of tumour recurrence and is regarded as a prerequisite for systemic tumour dissemination 25, 26. Our study revealed that TRIM4 expression levels in HCC tissues were negatively associated with vascular invasion and the absence of a tumour capsule, indicating that TRIM4 may be a potential prognostic marker for HCC.

For HBV-related HCC, the HKLC staging system is thought to exhibit good prognostic accuracy, and the use of this system is associated with high therapeutic efficacy 16, 27. In our cohort, 81.34% (109/134) of patients were positive for HBsAg. Therefore, our patients were categorized according to HKLC stage. We found that low TRIM4 expression in HCC is correlated with advanced HKLC stage. Therefore, we can propose that HCC cells with low TRIM4 protein levels may have higher invasive and metastatic potential, which promotes HCC progression and leads to a worse prognosis for HCC patients.

However, the molecular mechanisms by which TRIM4 regulates HCC progression must be further illuminated. Previous studies have revealed the role of some other TRIM family members in carcinogenesis or tumour progression. For example, TRIM29, which appears to be localized primarily to the cytoplasm, can bind to and stabilize Dvl-2; this interaction results in the release of β-catenin from the destruction complex and the activation of downstream β-catenin/TCF-regulated target genes and subsequently contributes to the development of pancreatic cancer 28. TRIM25, which plays a dual role in regulating the p53/Mdm2 circuit, can not only increase the abundance of p53 and Mdm2 by inhibiting their ubiquitination and degradation by 26S proteasomes but also inhibit the activity of the p53 protein 29, 30. Further research will provide evidence regarding whether an aberrant TRIM4 level can influence the Wnt and/or p53 signalling pathways.

In conclusion, our study revealed that TRIM4 expression levels in HCC tissues were an independent prognostic factor for OS and RFS in HCC patients. Close clinical monitoring is recommended for patients with low TRIM4 expression, and the biological functions of TRIM4 in regulating HCC progression require further elucidation.

## Figures and Tables

**Table 1:** Association of TRIM4 level with the Clinicopathological Parameters of 134 HCC Patients

Clinicopathological Parameters	Total	High TRIM4 expression		
		N (%)	χ2	*p* value
Sex	134			
Male	115	59(51.30%)	0.101	0.751
Female	19	9(47.37%)		
Age range, yr			2.971	0.085
≤ 57	69	40(57.97%)		
>57	65	28(43.08%)		
Tumor size, cm			1.883	0.170
≤5	73	41(56.16%)		
>5	61	27(44.26%)		
Tumor number			0.170	0.680
1	114	57(50.00%)		
≥2	20	11(55.00%)		
Grade of differentiation			0.088	0.767
I-II	100	50(50.00%)		
III-IV	34	18(52.94%)		
Vascular invasion			4.740	0.029
Yes	41	15(36.59%)		
No	93	53(56.99%)		
Tumor capsule			9.655	<0.01
None	61	22 (36.07%)		
Yes	73	46 (63.01%)		
HBsAg			0.093	0.761
Positive	109	56(51.38%)		
Negative	25	12(48.00%)		
Cirrhosis			2.502	0.114
Positive	74	33(44.60%)		
Negative	60	35(58.33%)		
AFP, ng/ml			0.448	0.503
≤400	85	45(52.94%)		
>400	49	23(46.94%)		
HKLC stage			4.507	0.034
Early	57	35(61.40%)		
Advanced	77	33(42.86%)		

**Table 2:** Univariate and multivariate analysis of prognostic factors of RFS in 134 HCC patients

Variable	Univariate		Multivariate	
	χ2	*p* value	HR (95%Cl)	*p* value
TRIM4 expression (high vs low)	12.190	<0.001	0.345-0.930	0.025
Tumor diameter (>5 cm vs ≤5 cm)	11.162	0.001	1.131-2.876	0.013
Vascular invasion (yes vs no)	14.189	<0.001	1.115-2.912	0.016
Tumor capsule (none vs yes)	4.726	0.030	0.798-2.091	0.298
Tumor number (≥2 vs 1)	0.991	0.320	-	n.a.
Sex (male vs female)	1.000	0.317	-	n.a.
Age (years) (≤57 vs>57)	0.110	0.740	-	n.a.
AFP (ng/ml) (≤400 vs >400)	2.264	0.132	-	n.a.
Tumor differentiation (III-IV vs I-II)	0.859	0.354	-	n.a.
Liver cirrhosis (yes vs none)	0.007	0.932	-	n.a.
HKLC stage (advanced vs early)	16.275	<0.001	-	n.a.

n.a., not applicable.

**Table 3:** Univariate and multivariate analysis of prognostic factors of OS in 134 HCC patients

Variable	Univariate		Multivariate	
	χ2	*p* value	HR (95%Cl)	*p* value
TRIM4 expression (high vs low)	8.874	<0.01	0.321-0.944	0.030
Tumor diameter (cm) (>5 vs ≤5)	14.497	<0.001	1.264-3.798	<0.01
Vascular invasion (yes vs no)	15.798	<0.001	1.322-3.826	<0.01
Tumor capsule (none vs yes)	0.385	0.535	-	n.a.
Tumor number (≥2 vs 1)	0.559	0.455	-	n.a.
Sex (male vs female)	0.308	0.579	-	n.a.
Age (years) (≤57 vs >57)	0.003	0.955	-	n.a.
AFP (ng/ml) (≤400 vs >400)	2.150	0.143	-	n.a.
Tumor differentiation (III-IV vs I-II)	1.411	0.235	-	n.a.
Liver cirrhosis (positive vs negative)	0.499	0.480	-	n.a.
HKLC stage (advanced vs early)	13.465	<0.001	-	n.a.

n.a., not applicable.

**Table 4:** Univariate and multivariate analysis of prognostic factors of RFS in 200 HCC patients

Variable	Univariate		Multivariate	
	χ2	*p* value	HR (95%Cl)	*p* value
TRIM4 expression (high vs low)	10.795	0.001	0.371-0.795	0.002
Tumor diameter (cm) (>5 vs ≤5)	0.130	0.719	-	n.a.
Vascular invasion (yes vs no)	2.707	0.100	-	n.a.
Tumor capsule (none vs yes)	1.744	0.187	-	n.a.
Tumor number (≥2 vs 1)	20.094	<0.001	1.620-3.713	<0.001
Sex (male vs female)	0.002	0.968	-	n.a.
Age (years) (≤50 vs >50)	1.020	0.313	-	n.a.
AFP (ng/ml) (≤400 vs >400)	0.163	0.686	-	n.a.
Tumor differentiation (III-IV vs I-II)	0.008	0.929	-	n.a.
Liver cirrhosis (positive vs negative)	3.090	0.079	-	n.a.

n.a., not applicable.

**Table 5:** Univariate and multivariate analysis of prognostic factors of OS in 200 HCC patients

Variable	Univariate		Multivariate	
	χ2	*p* value	HR (95%Cl)	*p* value
TRIM4 expression (high vs low)	9.004	<0.01	0.386-0.951	0.029
Tumor diameter (cm) (>5 vs ≤5)	1.338	0.247	-	n.a.
Vascular invasion (yes vs no)	8.596	<0.01	0.916-2.477	0.106
Tumor capsule (none vs yes)	1.866	0.172	-	n.a.
Tumor number (≥2 vs 1)	12.678	<0.001	1.240-3.185	<0.01
Sex (male vs female)	0.148	0.701	-	n.a.
Age (years) (≤50 vs >50)	0.722	0.395	-	n.a.
AFP (ng/ml) (≤400 vs >400)	5.018	0.025	0.699-1.948	0.556
Tumor differentiation (III-IV vs I-II)	0.434	0.510	-	n.a.
Liver cirrhosis (positive vs negative)	4.463	0.035	0.904-3.633	0.094

n.a., not applicable.

## Abbreviations

HCC: hepatocellular carcinoma
OS: overall survival
RFS: recurrence-free survival
TMAs: tissue microarrays
HBV: hepatitis B virus
AFP: alpha-fetoprotein
